# Bis(sulfosuccinimidyl) suberate (BS^3^) crosslinking analysis of the behavior of amyloid-β peptide in solution and in phospholipid membranes

**DOI:** 10.1371/journal.pone.0173871

**Published:** 2017-03-21

**Authors:** Jing-Ming Shi, Jie Pei, En-Qi Liu, Lin Zhang

**Affiliations:** 1 Research Institute of Atherosclerotic Disease, Xi’an Jiaotong University Cardiovascular Research Center, Xi’an P. R. China; 2 School of Medicine, Xizang Minzu University, Xian’ yang P. R. China; 3 Lanzhou Institute of Husbandry and Pharmaceutical Sciences, Chinese Academy of Agricultural Sciences, Lanzhou P. R. China; University of Hyderabad, INDIA

## Abstract

The structure and state of amyloid-β peptide (Aβ) oligomers often need to be checked by reliable experimental methods. Electrophoresis is a commonly applied measurement method. However, due to the presence of detergents, oligomers are easily broken during electrophoresis, which makes it very hard to accurately assess Aβ aggregate states. In the current study, bis(sulfosuccinimidyl) suberate (BS^3^) was used to cross-link Aβ1–42 oligomers prior to electrophoresis. When compared to a previously reported Aβ cross-linking agent, glutaraldehyde, it was quite apparent that BS^3^ is more suitable for detecting intra-membrane Aβ oligomers and extra-membrane Aβ oligomers states. As such, our findings provide an efficient method for analyzing Aβ proteins or other proteins that are easily aggregated in solution and in phospholipid membranes.

## Introduction

Alzheimer’s disease (AD) is the most common cause of dementia. Worldwide, there are 40 million people who have dementia, including 60–70% of AD cases, and this number is expected to double every 20 years [1]. The pathological changes in AD brain tissues include the extracellular deposition of neuritic plaques that are mainly composed of amyloid-β peptide(Aβ), as well as the intracellular formation of neurofibrillary tangles induced by hyperphosphorylated tau (p-tau). Although the pathogenesis of AD is unclear, after several decades of research, Aβ is still considered the main pathogenic factor for the disease [2,3]. Aβ in cerebrospinal fluid, total tau protein content and phosphorylated tau content have been used as biological markers and detected in clinical trials [4,5], and these indexes are indispensable in the prevention and treatment of AD.

Aβ is generated from amyloid precursor protein via sequential hydrolysis by β- and γ-secretases and rapidly forms soluble oligomers upon release. Aβ oligomers play critical toxic roles in the initial stage of AD [6]. These oligomers are not comprised of a single oligomer, but include various different oligomers.This is also the case for homogeneous Aβ-derived diffusible ligands (ADDL), which, by electron microscopy have been found to contain a mixture of oligomers ranging from 3-mers to 24-mers [7]. As such, it is difficult to determine which oligomers are responsible for Aβ toxicity. Therefore, assessing the ability of Aβ to aggregate is necessary to understand the mechanisms underlying its toxicity.

Electrophoresis is a frequently used method for detecting Aβ aggregates. During electrophoresis, the Aβ1–42 oligomer structure is damaged by the denaturing agents, such as sodium dodecyl sulfonate (SDS), and physically constrained by polyacrylamide gel electrophoresis (PAGE) channels. On electrophoresis gels, soluble Aβ oligomers usually display bands that correspond to three forms, monomers, trimers and tetramers [8]. Size exclusion chromatography (SEC) has been used to isolate low-molecular-weight Aβ oligomers and high-molecular-weight Aβ aggregates; moreover, SEC is one of the most commonly used methods for isolating and purifying Aβ oligomers[9,10], while its original aggregated form is unknown

Crosslinking helps to stabilize the structure of oligomers and can thus be used to reflect their native structure. Glutaraldehyde and photo-crosslinking have been used to analyze oligomers [11–13].

These experiments focused more on the results of cross-linking but did not pay attention to whether they were crosslinking monomers into oligomers. If the monomers are cross-linked into oligomers, the reaction is obviously overdone, and does not achieve the purpose of cross-linking. To achieve higher resolution, we tested the efficacy of bis(sulfosuccinimidyl)(BS^3^), a water-soluble compound that has previously been used in the cross-linking of antibodies [14,15]. BS^3^ has previously been used to cross-link intracellular epidermal growth factor receptor (EGFR) dimers, which shows that BS^3^ can be used as a cross-linking agent for membrane proteins [16]. Additionally, an established protocol for achieving the dimerization of EGFR via cross-linking by BS^3^has recently been reported [17].

This article reports on oligomeric studies of intra-membrane and extra-membrane Aβ1–42 using BS^3^ as a cross-linker. Our experimental studies will facilitate further studies of AD pathogenesis.

## Materials and methods

Aβ1–42 peptides were purchased from the company (Athens, GA, USA). 1,2-dipalmitoyl-sn-glycero-3-phosphatidylcholine semisynthetic (DPPC, purity> 99%) was purchased from Avanti Polar Lipids (Alabaster, AL). The BS^3^crosslinker was commercially available from Pierce (Pierce, USA). The mouse monoclonal antibody, 4G8, was purchased from Covance (Covance, USA), and ECL reagent was purchased from Millipore (Millipore, MA); 1,1,1,3,3,3-hexafluoro-2-propanol (HFIP) and dimethylsulfoxide DMSO were purchased from Sigma. F-12 medium was purchased from BioSource (Ham's F-12, BioSource, Australia). Thetransmission electron microscope was a Tecnai G20 (FEI, Netherlands).

### Aβ1–42 monomer treatment and oligomer preparation

A total of 222 μl HFIP was added to 1 mg Aβ, and the solution was mixed with a vortex for 5 min and left overnight at 4°C with slow shaking. The solution was then aliquoted into tubes containing 8.6 μl each, evaporated in N_2_ flow, and stored at -80°C. ADDL preparation was carried out as follows: F12 medium was added to an Aβ tube to give a final concentration of 100 μM, and the sample was incubated at 4°C for 24 hours [18]. Protofibrils preparation was carried out as follows: Aβ1–42 was added to Tris buffer (50 mM Tris, 100 mM NaCl, pH = 7.4) to a final concentration of 200 μM, and the solution was incubated with slow shaking at room temperature for 24 hours [6].

### Crosslinking and electrophoresis

First, 5 μM Aβ1–42 and 0.3 mM BS^3^ or 0.3 mM glutaraldehyde were added to PBS buffer, and the mixtures were allowed to crosslink in ice bath for varying durations. The reaction was then terminated with 1 M Tris (pH7.4). The samples were heated in 5×sample loading buffer at 90°C for 5 minutes before electrophoresis. During electrophoresis, a 4% stacking gel and a 16.5% Tris-tricine separation gel were used.

### Liposome preparation

First, 1 ml of phospholipids was vacuum-dried to remove solvents in nitrogen air overnight. Then, 1,000 μl PBS, which was preheated to 70°C, was added to the phospholipids, and the samples were incubated in a 70°C water bath after they were mixed with vortex oscillation. During incubation, the solution was removed from the water bath several times to be vortexed. After incubation, the solution was sonicated for 15 s and subjected to at least 5 freeze-thaw cycles. Liposomes were formed once the solution passed through a filter at temperatures above the phase-transition temperatures of the phospholipids [19].

### Transmission electron microscopy (TEM) sample preparation

First, 2 μl Aβ42 solution was added to discharge/hydrophilic-treated carbon films and incubated for 1 min. After washing with ddH_2_O, the carbon films were negatively stained with 1% sodium phosphotungstate for 1 min. Then, excess liquids were removed, and the carbon films were air-dried for TEM observation.

### SEC

SEC was carried out as previously described [20], with some modifications. A Biologic Quadtec UV-Vis Detector (Bio-Rad Laboratories, Hercules, CA, USA) and Superdex 75 10/300 GL column (Tricorn) were used. Columns were eluted at a flow rate of 0.5 mL/min. Aβ40 samples were centrifuged for 10 min at 15,000 × *g* and 4°C), and the supernatant (50 μL) was then injected into the column. Samples were fractionated at a flow rate of 0.75 mL/min, and peptides were detected by measuring the UV absorbance at 210 nm. Each experiment was performed at least three times.

## Results

### Principle of the BS^3^ crosslinking reaction

**Fig 1:**
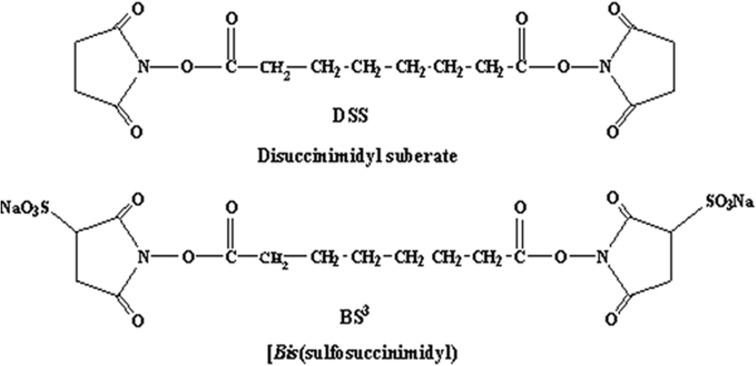
Molecular structures of BS^3^. Molecular structures of di-succinimidylsuberate (disuccinimidyl suberate, DSS) and bis-succinimidylsuberate sodium salt (Bis(sulfosuccinimidyl, BS^3^).

Di-succinimidylsuberate(DSS) is a water-insoluble N-hydroxysuccinimide ester (NHS-ester), and BS^3^, shown in Fig 1, is its water-soluble analog.

Both of them are α-amine-reactive, and although α-amines present on the N-termini of peptides react with NHS-esters, they are seldom available on proteins. Therefore, the reaction with side chains of peptides is the majorreaction that occurs. Furthermore, while five amino acids have nitrogen in their side chains, only the **ε**-amine of lysine will react to produce a stable product. When BS^3^ dissolved, its N-containing portion is rapidly removed, resulting in α-amino acids or lysine at the two terminals of this compound. The NHS-Ester Reaction Scheme of amino acids crosslinking with BS^3^ is described as S1 Fig.

Aβ1–42 has two residues, Lysine-16 and Lysine-28, which can be crosslinked to BS^3^. The hydrolysis reaction is a competitive reaction and can be terminated by 1 M Tris HCl or 1 M Glycine solution. This NHS-Ester Reaction (S1 Fig) has been previously described in the literature [17,21,22].

In this study, BS^3^ was adopted to cross-link Aβ1–42. The results are as follows.

### Controlling the crosslinking of Aβ monomers by BS^3^

**Fig 2:**
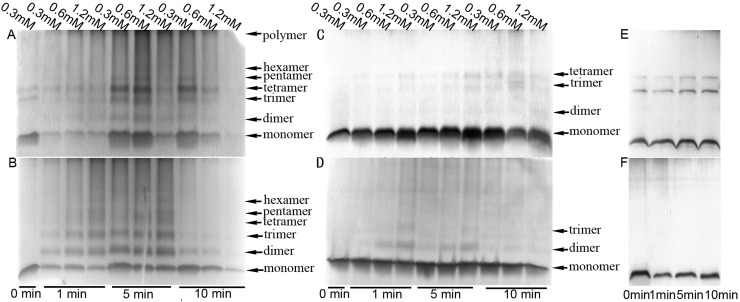
Aβ monomers cross-linked with glutaraldehyde and BS^3^. (A) Aμ1–42 and (B) Aμ1–40 monomer cross-linked with glutaraldehyde under ice bath conditions. The final concentrations of glutaraldehyde were 0.3 mM, 0.6 mM and 1.2 mM, and 1 minute, 5 minute and 10 minute incubation periods were used. (C) Aμ1–42 and (D) Aμ1–40 monomer cross-linked with BS^3^ under ice bath conditions. The final concentration of BS^3^ were 0.3 mM, 0.6 mM and 1.2 mM, and 1 minute, 5 minute and 10 minute incubation periods were used. A 5 μM final concentration of Aβ was used in all the glutaraldehyde and BS^3^ crosslinking experiments. A total of 1 μg sample was added to each electrophoresis lane, and the 16.5%Tris-Tricine gel was analyzed using silver staining. The concentration of Aβ42 (E) and Aβ40 (F) were the same as the crosslinking concentration. Samples were incubated in an ice bath for 1, 5, or 10 min.

According to previous reports, membrane-interacting Aβ1–40 can be crosslinked with 12 mM glutaraldehyde for 10 minutes [11]. To avoid excessive crosslinking, we used significantly lower concentration gradients of 0.3 mM, 0.6 mM and 1.2 mM and much shorter time gradients in an ice bath. The Aβ concentration was kept at 5 μM. The result showed that the Aβ1–40 and Aβ1–42 monomer could also readily form several oligomeric forms (Fig 2A, Fig 2B).

The ability of glutaraldehyde and BS^3^ to crosslink the Aβ1–42 monomer was compared for different concentration gradients and time gradients. The results are shown in Fig 2. When the lowest concentration (0.3 mM) and short crosslinking duration (1 minute) was used, glutaraldehyde crosslinked monomers into oligomers, whereas BS^3^ did not result in excessive crosslinking, even after a crosslinking duration of 5 minute. However, different concentrations of glutaraldehyde and BS^3^ barely affected crosslinking. It can be seen from Fig 2D that crosslinking was weaker when 1.2 mM of crosslinkers was used, as compared to when 0.6 mM of crosslinkers was used (refer to the discussion section).

### BS^3^ is more sensitive for Aβ oligomeric studies, compared to glutaraldehyde, as a crosslinker

**Fig 3:**
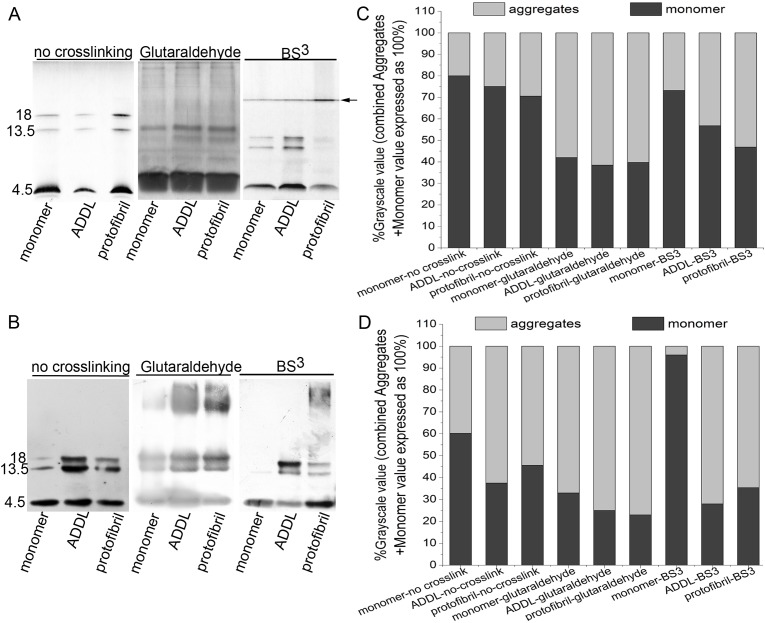
Aβ1–42 monomers, ADDL and protofibrils were cross-linked with glutaraldehyde or BS^3^. All experiments were carried out under ice bath conditions. The samples were analyzed on a 16.5% Tris-tricine gel. (A) In the absence of crosslinking, three forms of Aβ1–42. Aβ1–42 was crosslinked with 0.3 mM glutaraldehyde for 1 minute.Aβ1–42 was cross-linked with 0.3 mM BS^3^ for 5 minutes, and 1 μg sample was added to each electrophoresis lane. The gels were analyzed using silver staining. The "←" indicates the position of the aggregates in the protofibril lane. (B) The same crosslinking reaction conditions as “(A)” were used. But in "(B)", the amount of protein used in each electrophoresis lane was 10 ng. The results were analyzed using the murine monoclonal antibody, 4G8. (C) Histogram showing the grayscale value of each band in (A). “Aggregates” indicates the sum of all other bands excluding monomers. (D) Histogram showing the grayscale value of each band in (B).

ADDL and protofibril are the two most common Aβ1–42 aggregates [6]. Aβ monomers, ADDL and protofibril were analyzed with a 16.5% Tris-tricine gel. As shown in Fig 3. In the absence of crosslinking, the gray value percentages of aggregate bands were determined in monomers, ADDL, and protofibril. Aggregates of monomers showed reduced percentages by silver staining and western blotting. However, the numbers of Aβ1–42 bands did not differ significantly for the three forms. When 0.3 mM glutaraldehyde was used to crosslink the Aβ1–42 monomer, ADDL, and protofibrils for 1 min, this reagent caused excessive crosslinking overall, and therefore, it was difficult to distinguish the differences among the three Aβ1–42 samples based on the gray values and band numbers. After crosslinking with 0.3 mM BS^3^ for 5 min, different Aβ1–42 forms, which showed contrasting electrophoresis bands, were present. The monomer and the ADDL sample showed monomer, trimer and tetramer bands when silver staining and western blotting, the protofibril sample showed monomer, trimer, tetramer and higher oligomer bands; for protofibril, the percentage of tetramers and trimers was reduced, while that of the higher oligomers was significantly increased. There were also major differences in the percentages of aggregate gray values between the three samples. Therefore, as a crosslinker, BS^3^ was much better in distinguishing different oligomers in Aβ samples, as compared to glutaraldehyde. Additionally, even higher oligomers may have been present.

**Fig 4:**
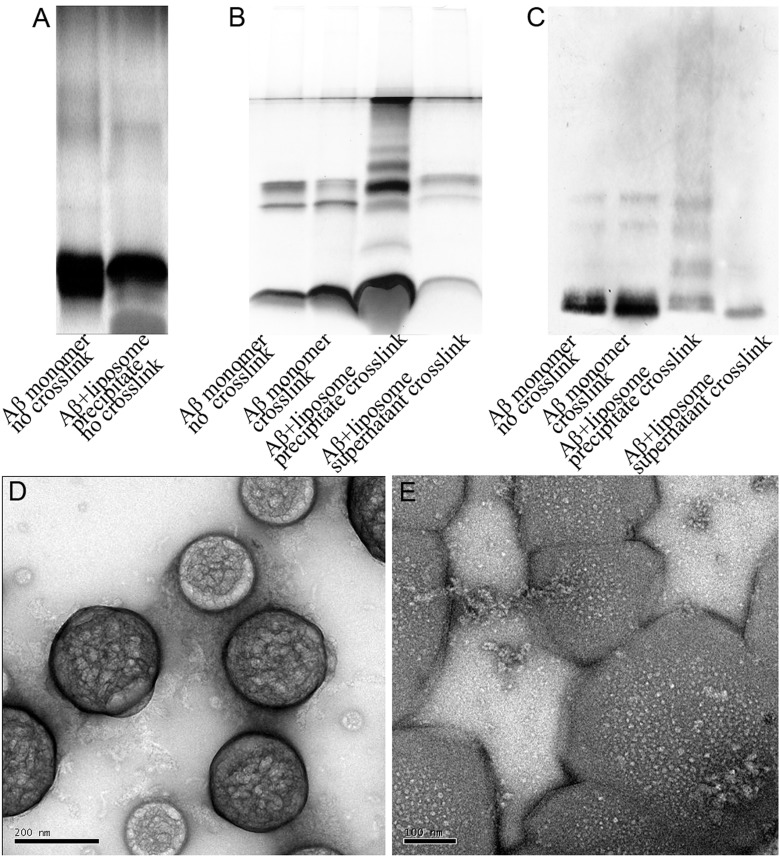
BS^3^ crosslinking to analyze the behavior of Aβ1–42 interaction with liposomes **(A)** The Aβ1–42 monomer was incubated with liposomes in the absence of crosslinking. The samples were assessed by electrophoresis and silver stain analysis. There was no significant difference when comparing the Aβ1–42 monomer with "Aβ1–42 monomer + liposome". (B) The Aβ1–42 monomer was added to liposomes, and the mixture was crosslinked with 3 mM BS^3^ for 5 minutes under ice bath conditions. Compared with the Aβ1–42 monomer, the "Aβ1–42 monomer + liposome" precipitate, but not the supernatant samples, clearly formed oligomers. A total of 1 μg of sample was added to each electrophoresis lane. (C) All experimental conditions were the same as "B", but the amount of protein used in each electrophoresis lane was 10 ng. The results were analyzed by a murine monoclonal antibody, 4G8, and Western blot. (D) Aβ1–42 DPPC was incubated with liposomes for 2 hours. The staff was 200 nm, and the liposomes had a diameter of 300 nm. After discarding the Aβ1–42 fiber, the samples were centrifuged and resuspended. The samples without crosslinking were assessed by TEM. (E) All experimental conditions were the same as "(D)", but the sample was crosslinked with 3 mM BS^3^ before TEM sample preparation. The staff was 100 nm.

Previous studies have shown that Aβ1–42 oligomerization can be induced through membrane insertion or membrane adsorption. Aggregation mechanisms include membranes as a reaction platform to accelerate Aβ aggregation, electrostatic effects, and Aβ conformational changes, resulting in the formation of ion channels [8,23–25]. In order to analyze the state of Aβ1–42 when interacting with a membrane, Aβ1–42 was added to a DPPC liposome solution. A final concentration of 1 μM Aβ1–42 monomer was used, and the mixture was incubated for two hours at room temperature. After centrifugation at 15,000 g × 10 min to remove large aggregates (fibers), the resulting supernatant was analyzed using Transmission electron microscopy (TEM), as shown in Fig 4D and 4E(more seen S3 Fig).The supernatant was subjected to additional ultracentrifugation step at 200,000 g × 30 min. In the samples in which no crosslinking had occurred, the electrophoretic bands for the Aβ1–42 monomers and the precipitants of the Aβ1-42-liposome showed no significant difference, as shown in Fig 4A. For the samples that had been crosslinked with BS^3^, the precipitant and supernatant of the Aβ1-42-liposome displayed different bands in SDS-PAGE, as indicated in Fig 4B (silver staining) and 4C (Western Blot analysis). Compared to non-crosslinked cases, BS^3^ crosslinking produced a significant amount of oligomeric Aβ1–42. The same conclusion was reached based on the silver staining and Western Blot analysis. Taken together, these results suggest that as compared to glutaraldehyde, using as a crosslinker BS^3^ is applicable not only to different oligomeric states of Aβ1–42, but also to Aβ1–42 in the context of a membrane.

### BS^3^-Aβ1–42 crosslinking observed by TEM

**Fig 5:**
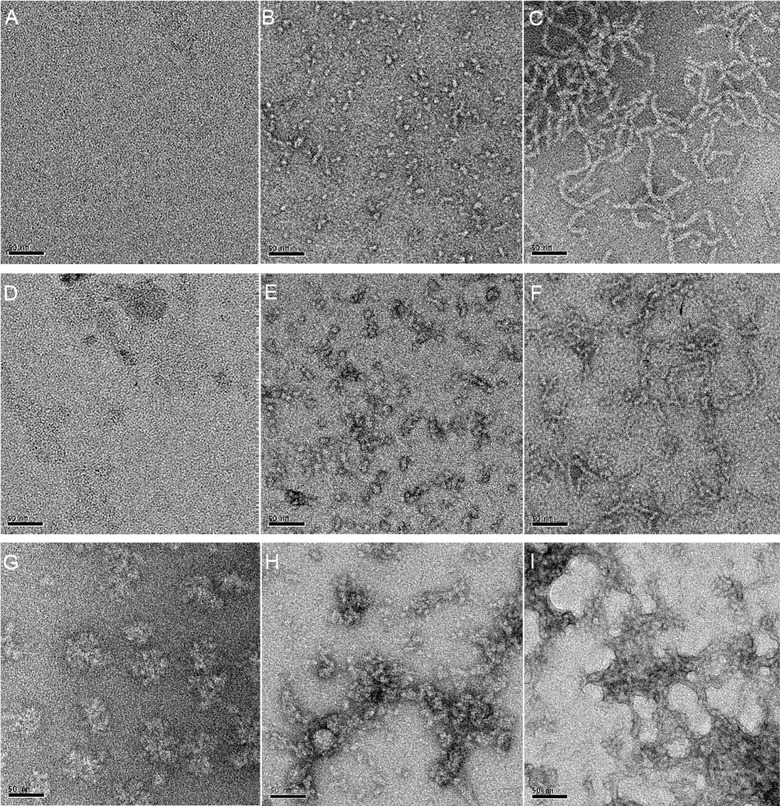
Electron microscope photographs of Aβ1–42 monomer, ADDL and protofibril. (A), (B) and (C) show the non-crosslinked samples. (D), (E) and (F) show the samples that had been crosslinked with 0.3 mM BS^3^ for 5 minute. (G), (H) and (I) are samples that had been crosslinked with 0.3 mM glutaraldehyde for 1 minute. The staff was 50 nm.

The Aβ1–42 monomer, ADDL and protofibril were assessed by TEM. The Aβ1–42 monomer was only one nanometer, and it was difficult to distinguish the non-crosslinked form from that which had crosslinked with BS^3^ (Fig 5A and 5D). Compared to Fig 5E and 5F, Fig 5G and 5H showed considerable Aβ1–42 aggregates after crosslinking with BS^3^. When glutaraldehyde was used, severe crosslinking occurred. In contrast, BS^3^ caused only mild crosslinking of Aβ1–42, and these conclusions are similar to those obtained from the electrophoresis results.

### Analysis of BS^3^-Aβ crosslinking by SEC

Aβ monomers and aggregates exist simultaneously in nature before and after crosslinking. SDS-PAGE was used to characterize these monomers and aggregates, as described above. However, since many Aβ mixtures are SDS-labile, the use of SDS-PAGE can lead to some problems in the analysis. Accordingly, the use of SEC combined with SDS-PAGE may be appropriate because SEC fractionates Aβ samples under nondenaturing conditions on the basis of molecular weight. SEC has been used to analyze different aggregation states of Aβ in solution [20,26].

**Fig 6:**
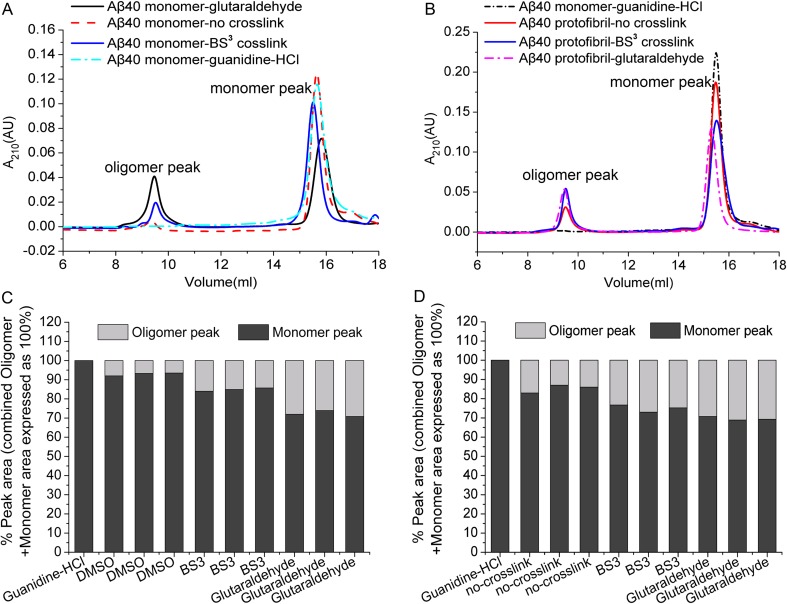
Analysis of BS^3^-Aβ crosslinking by SEC on a Superdex 75 10/300 GL SEC column. (A) Aβ40 monomer (red line: DMSO solubilization; dark blue line: 6 M guanidine-HCl solubilization; sapphire line: 0.3 mM BS^3^ crosslinking; black line: 0.3mM glutaraldehyde crosslinking). Fifty microliters was used for each sample (Aβ40 concentration: 150 μM). (B) Aβ40 protofibrils (red line: no crosslinking; sapphire line: 0.3 mM BS^3^ crosslinking; pink line: 0.3 mM glutaraldehyde crosslinking). Aβ40 monomer (black line: 6 M guanidine-HCl solubilization). Fifty microliters was used for each sample (Aβ40 concentration: 300 μM). (C) Analysis of the peak area for the elution of monomeric Aβ40 (crosslinked or not crosslinked) from monomer samples. Each sample was injected three times. For each sample analysis, the total (aggregate + monomer) peak area was expressed as 100%. For Aβ40 monomers without crosslinking, the monomer:aggregate ratio was 93%:7%. For Aβ40 monomers with BS^3^ crosslinking, the monomer:aggregate ratio was 84.87%:15.13%. For Aβ40 monomers with glutaraldehyde crosslinking, the monomer:aggregate ratio was 72%:28%. (D) Analysis of the peak area for the elution of protofibril Aβ40 (crosslinked or not crosslinked) from monomer samples. Each sample was injected three times. For each sample analysis, the total (aggregate + monomer) peak area was expressed as 100%. For Aβ40 protofibrils without crosslinking, the monomer:aggregate ratio was 85.3%:14.7%. For Aβ40 protofibrils with BS^3^ crosslinking, the monomer:aggregate ratio was 75%:25%. For Aβ40 protofibrils with glutaraldehyde crosslinking, the monomer:aggregate ratio was 69.6%:30.4%.

Glutaraldehyde- and BS^3^-crosslinked Aβ samples showed different peak shapes from SECunder nondenaturing conditions. The Aβ40 monomer and protofibrils were treated with 6 M guanidine hydrochloride, DMSO, 0.3 mM glutaraldehyde, or BS^3^ and analyzed on Superdex 75 10/300 GL columns. The results are shown in Fig 6. Aβ40 monomer samples treated with guanidine hydrochloride had only one monomer peak, whereas the DMSO-treated monomer sample showed two oligomer peaks (one small and one large). In the absence of crosslinking, the Aβ40 oligomer sample showed a monomer peak and an oligomer peak. The Aβ40 agglutination diversity pattern was not separated, consistent with previous reports showing that low-molecular-weight oligomers are in rapid equilibrium with monomers and cannot be distinguished [27,28]. Glutaraldehyde-crosslinked monomer and oligomeric samples showed oligomeric peak area ratios that were larger than those of BS^3^-crosslinked samples. For each injection, the total (monomer + aggregate) peak area was expressed as 100%. For monomer samples, the glutaraldehyde-crosslinked oligomeric peak area was 27.77%, whereas the BS^3^-crosslinked oligomeric peak area was 15.25%. For oligomeric samples, the glutaraldehyde-crosslinked oligomeric peak area was 30.37%, whereas the BS^3^-crosslinked oligomeric peak area was 25.03%. These data suggested that the intensity of glutaraldehyde crosslinking was stronger than that of BS^3^, consistent with the above electrophoresis and TEM data.

Based on the above results, BS^3^ is very useful for Aβ1–42 electrophoresis and electron microscopy analysis in the following aspects: 1) the moderate crosslinking of Aβ1–42 by BS^3^ helps identify different oligomers in Aβ monomers, ADDL and other forms aggregates through electrophoresis; 2) Compared to glutaraldehyde as a crosslinker, BS^3^ is more appropriate, as it does not lead to excessive crosslinking; 3) BS^3^ can be used to crosslink Aβ in the context of a membrane.

## Discussion

Soluble Aβ oligomers play an important role in the etiology of the AD process, and thus, in recent decades, the toxic effects of Aβ oligomers have been the subject of considerable research [29,30]. The study of Aβ often requires the detection of Aβ aggregates in order to distinguish monomeric, oligomeric or protofibrilic protein status, which is technically challenging. This same problem exists in the studies of other proteins prone to aggregation, such as α- synuclein, islet amyloid polypeptide (IAPP), human insulin and prion peptide [30,31]. in this paper, we assessed a method of oligomer detection using two common Aβ aggregates, ADDL and protofibril [7], as examples.

Electrophoresis analysis is one of the most commonly used methods in Aβ aggregate detection. However, the use of a denaturing gel results in altered Aβ oligomers, which no longer reflect the original aggregates. Glutaraldehyde has previously been used as a crosslinking agent, such as in the preparation of a decellularized vascular matrix by co-crosslinking procyanidins and glutaraldehyde [32]. However, when glutaraldehyde is used, the excessive cross-linking of Aβ occurs easily, even at very low concentrations and with short crosslinking times [33].

When glutaraldehyde was used, we observed severe aggregation, even for Aβ monomers (Fig 2 and S2 Fig), thus making it hard to differentiate the Aβ oligomer states. When BS^3^ was used, the crosslinking was relatively "mild", and excessive crosslinking of the Aβ1–42 monomer could be prevented, as long the process was kept to under 5 minutes, as shown in Figs 2 and 3. According to Fig 2, the crosslinking was weaker when the concentration of BS^3^ was increased to 1.2 mM and the duration was increased to 10min. This is because the large aggregates that had formed at higher BS^3^concentrations did not enter the gel during electrophoresis. Additionally, the intermediate portion of the gel was advantageous in silver staining, to a certain extent, since the edge strip dye was easily eluted when the entire acrylamide gel, which was placed in a Petri dish, was incubated with rocking on a rotary shaker during the dyeing-destaining process. The concentration of protein used in this study was 5 μM. If a higher protein concentration is required, a corresponding proportional increase in the concentration of BS^3^ would be required.In the experiments with liposomes, because the preliminary experiments showed that lipids have a greater tendency for interfering with crosslinking, BS^3^ was added to a final concentration of 3 mM in phospholipid membranes which was ten times of the concentration of BS^3^ in solution, as shown in Fig 4. Preliminary experiments using different concentration proportion of Aβ1–42 and phospholipid did not yield significantly different results.

Treatment of Aβ1–42 with glutaraldehyde appeared to induce the formation of trimers and tetramers, whereas treatment of Aβ1–42 with BS^3^ induced the formation of dimers and trimers. This may be due to the generation of different forms of aggregates induced by excessive crosslinking of glutaraldehyde; these aggregates may become more stable trimers and tetramers by extrusion through the electrophoresis gel pores. This concept of gel-induced oligomerization has been reported in a previous work [8] and can be explained by the ability of glutaraldehyde to produce the acetal reaction easily following self-aggregation, resulting in trimers and tetramers. BS^3^ is the most likely to remove the N group at both ends, thereby producing two dimers through the action of two Aβ molecules. One of the two dimers forms through Lys and then interacts with a BS^3^ molecule to form a trimer.

Our results showed that the trimers and tetramers in ADDL and protofibril samples that had not been crosslinked were more abundant than those in the monomer sample. This result could be explained by the observation that the larger aggregates may become more stable trimers and tetramers by extrusion through electrophoresis gel pores [8]. When BS^3^ was used as a crosslinker, few tetramers were found in the protofibril samples because BS^3^ stabilized the aggregates without inducing the formation of tetramers.

Gel electrophoresis was performed under denaturing conditions. However, SEC analyzes the crosslinking of proteins under nondenaturing conditions. Our results also showed that glutaraldehyde had a higher crosslinking capacity than BS^3^. Notably, in the SEC experiment, we did not use Aβ1–42 because Aβ1–42 is aggregates easily and cannot be easily maintained in the monomer state. Additionally, in UA210, the light absorption value is low, resulting in prevention of protein waste. This further supported the use of Aβ1–40 in this study. We did not use Aβ-40 ADDL because ADDL is prepared in culture medium, which contains many light-absorbing groups(S4 Fig). Therefore, in this study, we used Aβ1–40.

In short, for Aβ, which are prone to aggregate, electrophoresis after BS^3^ crosslinking enables the detection of discrete protein bands that correspond to different aggregates of Aβ. This suggests that BS^3^ crosslinking suitable for oligomer detection of Aβ from various sources, including chemically synthesized, naturally expressed and liposome-interacting Aβ. Therefore, BS^3^ crosslinking should be useful in the study of the toxicity mechanism of Aβ and AD.

## Supporting information

S1 Fig The NHS-Ester Reaction Scheme of amino acids crosslinking with BS3. (TIF)

S2 Fig Aβ1–42 monomers, ADDL and protofibrils were cross-linked with glutaraldehyde or BS3. (TIF)

S3 Fig Different Aβ1–42 forms and liposome samples were observed by TEM. (TIF)

S4 Fig Analysis of Aβ1–42 monomer, ADDL, and F12 culture medium by SEC. (TIF)
