# Supplementary figures and images for: Bis(sulfosuccinimidyl) suberate (BS3) crosslinking analysis of the behavior of amyloid-β peptide in solution and in phospholipid membranes
Source: PLoS One. 2017 Mar 21;12(3):e0173871. doi: 10.1371/journal.pone.0173871 (PMC5360245; doi:10.1371/journal.pone.0173871)

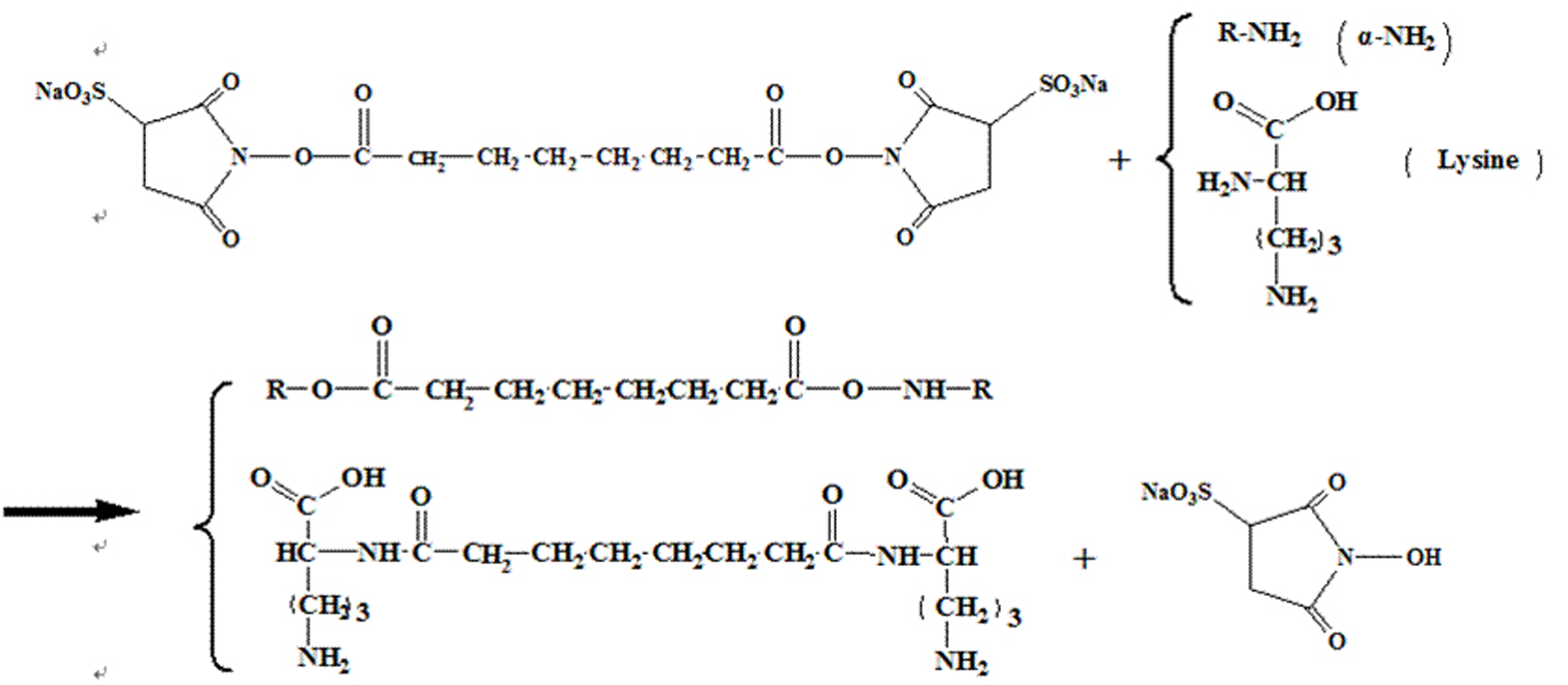

Supplement: S1 Fig — (TIF) [file pone.0173871.s001.tif]

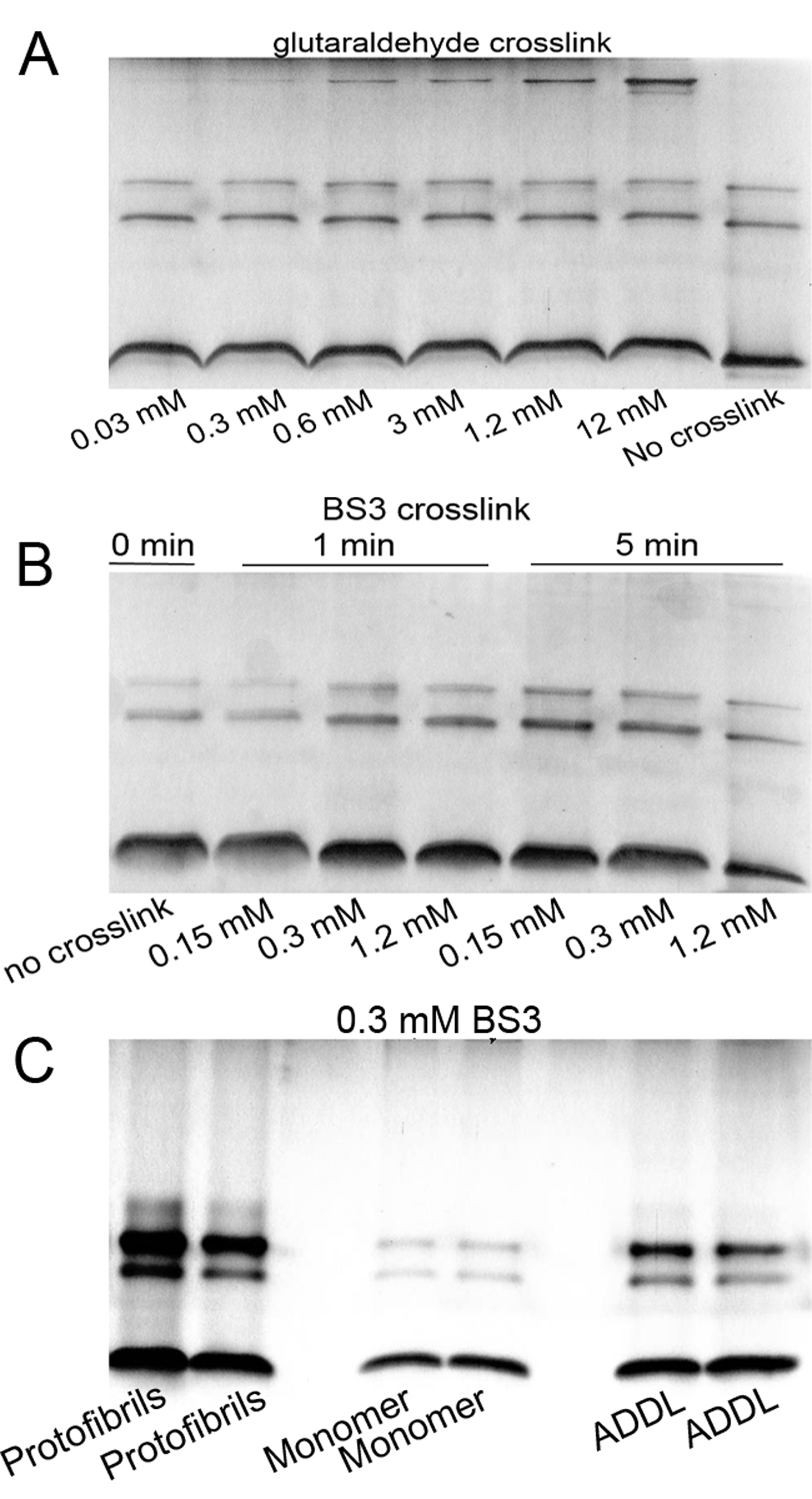

Supplement: S2 Fig — (TIF) [file pone.0173871.s002.tif]

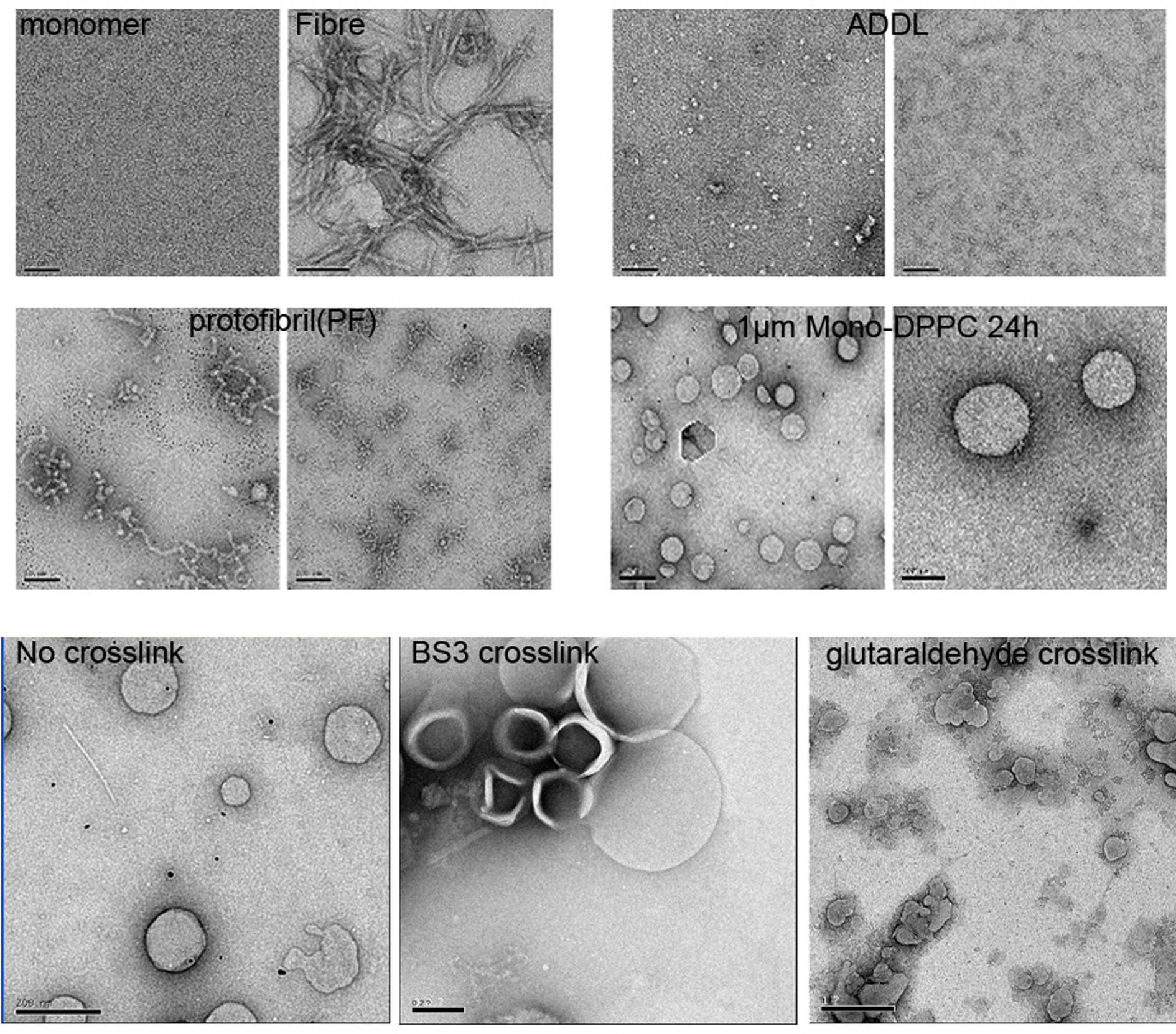

Supplement: S3 Fig — (TIF) [file pone.0173871.s003.tif]

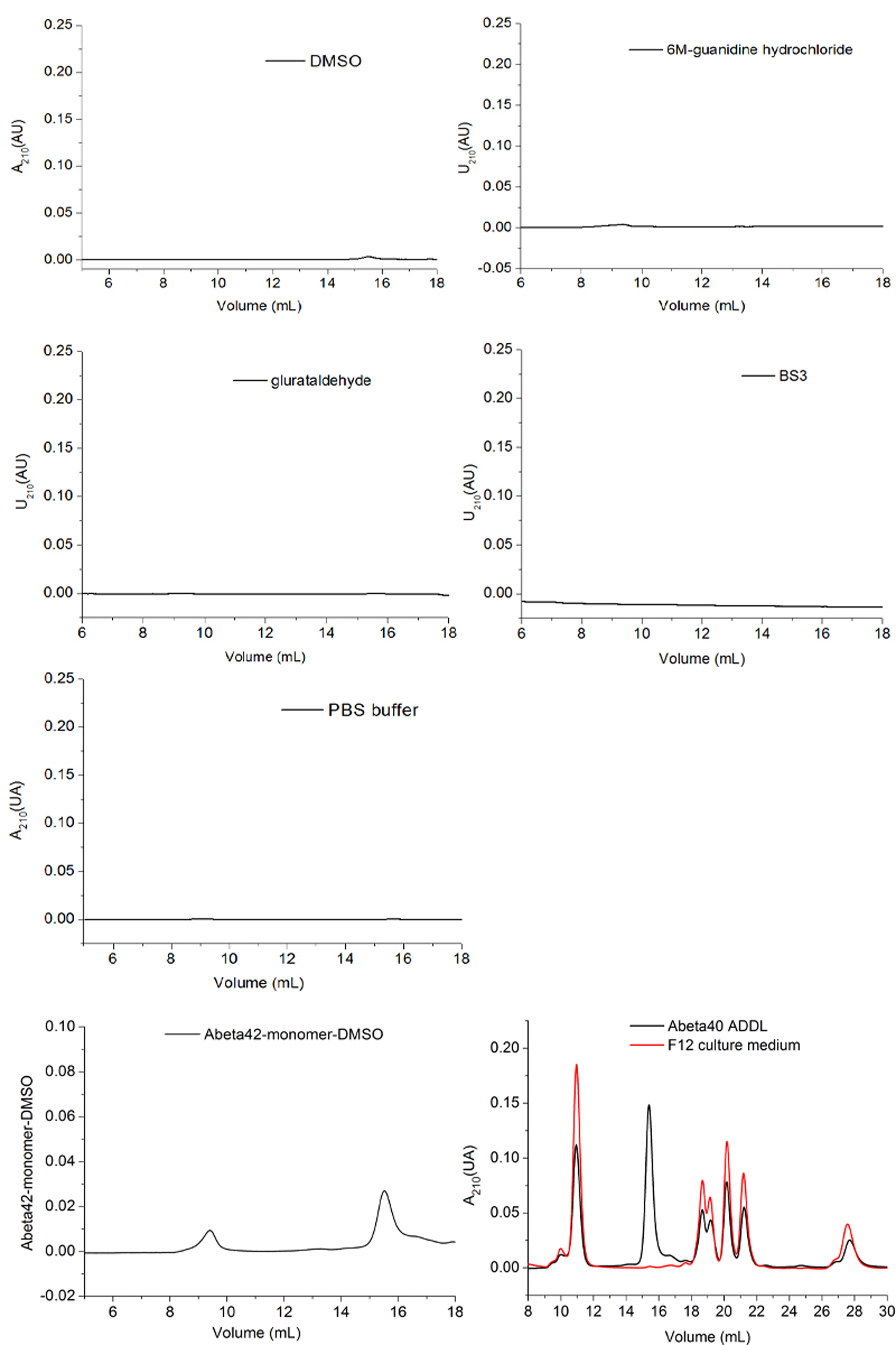

Supplement: S4 Fig — (TIF) [file pone.0173871.s004.tif]
